# Supplementary material for: Guillain-Barré Syndrome with Respiratory Failure following Spine Surgery for Incomplete Cervical Cord Injury: A Case Report and Literature Review
Source: Medicina (Kaunas). 2022 Aug 6;58(8):1063. doi: 10.3390/medicina58081063 (PMC9415430; doi:10.3390/medicina58081063)
Supplement: Supplementary file 1 [file medicina-58-01063-s001.zip › medicina-1808624-supplementary.pdf]

Supplementary Material

Table S1. Laboratory Data With Normal Value

Laboratory studies	Results	Normal value
Blood		
Total protein (g/dL)	5.7	6.0-8.0
ALKP (U/L)	127	50 – 190
AST (U/L)	14	8 – 38
ALT (U/L)	13	10 – 50
LDH (U/L)	224	120 – 240
Sodium (mEq/L)	142	137 - 153
Calcium (mg/dL)	8.9	8.4 – 10.2
Potassium (mEq/L)	4.6	3.5 – 5.3
Blood urea nitrogen (mg/dL)	12	5 – 25
Creatinine (mg/dL)	0.68	0.7 – 1.4
C reactive protein (mg/dL)	0.253	< 0.3
Lactate (mg/dL)	7.0	3 - 12
Glucose (mg/dL)	220	70 - 110
HbA1c (%)	7.2	4 – 5.6
WBC (/L)	7920	3900 - 10600
RBC ($x10^6/\mu L$)	3.77	4.5 - 5.9
Hemoglobin (g/dL)	11.2	13.5 - 17.5
Platelet ($x10^3/\mu L$)	232	150 - 400
Neutrophil (%)	67.6	40 - 74
Lymphocyte (%)	22.4	19 - 48
Monocyte (%)	7.7	3.4 - 9.0
Cerebrospinal fluid (CSF)		
Appearance	Colorless, Clear	Colorless, Clear
Specific gravity	1.008	1.003-1.009
RBC (/μL)	3	0 - 5
Total Nucleated Count (/μL)	0	0 - 5
Cytology	Negative for malignant cell.	
Total Protein (mg/dL)	167.3	15 - 40
Glucose (mg/dL)	73	50 - 75
Lactate (mg/dL)	20.7	10 – 25
Oligoclonal bands	Negative	Negative
IgG index	0.766	0.34 - 0.58
Microbiology		
Bacterial culture (blood, urine, Stool, CSF)	No growth for ordinary & anaerobic culture.	
Gram Stain (CSF)	Negative	
Viral Serology	Negative	
Influenza viral antigen test	Negative	
